# Approximate Bayesian computation and ecological niche models elucidate the demographic history and current fragmented population distribution of a Korean endemic shrub

**DOI:** 10.1002/ece3.10792

**Published:** 2023-12-06

**Authors:** Homervergel G. Ong, Yong‐In Kim, Jung‐Hoon Lee, Bo‐Yun Kim, Dae‐Hyun Kang, Eui‐Kwon Jung, Jae‐Seo Shin, Young‐Dong Kim

**Affiliations:** ^1^ Multidisciplinary Genome Institute Hallym University Chuncheon South Korea; ^2^ On Biological Resource Research Institute (OBRRI) Chuncheon South Korea; ^3^ National Institute of Biological Resources (NIBR) Incheon South Korea; ^4^ Korea National Park Research Institute Wonju South Korea; ^5^ Department of Life Science Hallym University Chuncheon South Korea

**Keywords:** *Abeliophyllum distichum*, Korean Peninsula, paleodistribution, phylogeography, white forsythia

## Abstract

Climatic fluctuations and geological events since the LGM are believed to have significantly impacted the population size, distribution, and mobility of many species that we observe today. In this paper, we determined the processes driving the phylogeographic structure of the Korean endemic white forsythia by combining the use of genome‐wide SNPs and predicting paleoclimatic habitats during the LGM (21 kya), Early Holocene (10 kya), Mid‐Holocene (6 kya), and Late Holocene (3 kya). Using a maximum of 1897 SNPs retrieved from 124 samples across nine wild populations, five environmental predictors, and the species' natural occurrence records, we aimed to infer the species' demographic history and reconstruct its possible paleodistributions with the use of approximate Bayesian computation and ecological niche models, respectively. Under this integrated framework, we found strong evidence for patterns of range shift and expansion, and population divergence events from the onset of the Holocene, resulting in the formation of its five distinct genetic units. The most highly supported model inferred that after the split of an ancestral population into the southern group and a larger central metapopulation lineage, the latter gave rise to the eastern and northern clusters, before finally dividing into two sub‐central groups. While the use of molecular data allowed us to identify and refine the (phylo)genetic relationships of the species' lineages and populations, the use of ecological data helped us infer a past LGM refugium and the directions of post‐glacial range dynamics. The time frames of these demographic events were shown to be congruent with climatic and geological events that affected the central Korean Peninsula during these periods. These findings gave us a better understanding of the consequences of past spatiotemporal factors that may have resulted in the current fragmented population distribution of this endangered plant.

## Graphical abstract

In this paper, we determined the processes driving the phylogeographic structure of the Korean endemic white forsythia by combining the use of genome‐wide SNPs and hindcasting of paleoclimatic habitats during the LGM (21 kya), Early Holocene (10 kya), Mid‐Holocene (6 kya), and Late Holocene (3 kya). Using a maximum of 1897 SNPs retrieved from 124 samples across nine wild populations, and five environmental predictors and the species' natural occurrence records, we aimed to infer the species' demographic history and reconstruct its possible paleodistributions with the use of approximate Bayesian computation and ecological niche models, respectively. Under this integrated framework, we found strong evidence for patterns of range shift, and population expansion/divergence events from the onset of the Holocene, resulting in the formation of its five distinct genetic units.

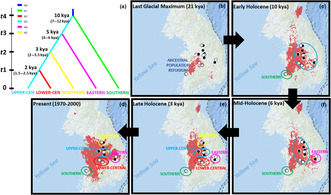

## 1 INTRODUCTION

Past climatic and geological events have affected the population size, spatial organization, and mobility of many species that we observe today. The most recent events that have made significant impacts on extant species and their distribution are perhaps those which occurred in the Quaternary, specifically the environmental changes since the last glacial maximum (LGM) ca. 21,000–18,000 years ago (Comes & Kadereit, 1998; Hewitt, 2004; Kimura et al., 2014; Petit et al., 2003; Qiu et al., 2011). Climate‐associated oceanic regression and transgression are also often attributed to having critically influenced species survival and distribution, and impacted genetic diversity, demographic history, and subsequent evolution (Kadereit & Westberg, 2007; Park et al., 2019; Weising & Freitag, 2007). In consideration of these past spatiotemporal phenomena, the current spatial arrangements of many plants are widely agreed to have been established since the onset of suitable environmental conditions after the LGM. For instance, paleodistribution studies on plant species with relatively limited occurrences suggested their survival during the harshly cold LGM conditions (e.g., Escobar et al., 2021; Gugger et al., 2013; Kimura et al., 2014), while those of other taxa even inferred expansion from small refugial areas throughout the climatic oscillations of the succeeding Holocene (e.g., Bagley et al., 2020; Ribeiro et al., 2019; Sakaguchi et al., 2010).

The effects of paleoclimatic fluctuations and oceanic regression‐transgression on the genetic structure and distribution of organisms, however, vary among geographical regions (Hewitt, 2000, 2004). While the consequences of past spatiotemporal events on vegetation in most parts of the Northern Hemisphere are well documented (Bagley et al., 2020; Bennett et al., 1991; Gugger et al., 2011; Ren et al., 2017; Soltis et al., 1997), the changes in climate and sea levels in East Asia are relatively less understood, simply because the region remained free from extended ice sheets during the glacial periods (Clark et al., 2009; Clark & Mix, 2002; Ehlers & Gibbard, 2007). Owing to the relatively stable environmental conditions in the region, some parts of East Asia are assumed to have served as glacial refugia that supported the survival of many plant species throughout the Quaternary climate change (Chung et al., 2018; Clark & Mix, 2002; Ehlers & Gibbard, 2007; Tang et al., 2018, 2020).

Investigating the effects of spatiotemporal changes on plant species distribution in East Asia is crucial to the further understanding of their present occurrence range, diversity, survival, and evolutionary dynamics. Regional studies revealed that as a consequence of the lowered sea level during the LGM, the exposed continental shelf that connected East China, the Korean Peninsula (KP), and South Japan (i.e., areas presently covered by the East China Sea, Yellow Sea, and East Sea/Sea of Japan, respectively) served as a refuge and at the same time a dispersal corridor to the ancestral populations of widely distributed East Asian plant species (Clark et al., 2019; Sakaguchi et al., 2012). Using a combined approach with paleoclimatic niche modeling, some of these regional investigations were able to infer paleoclimatically suitable habitats and glacial refugia, and even reconstruct patterns of post‐glacial migrations (Kimura et al., 2014; Qi et al., 2012; Sakaguchi et al., 2010, 2011; Worth et al., 2013).

The amount of research work that investigated the paleodistributions of native plants occurring on the KP, however, is still relatively small, especially those that integrated molecular methods in their design. When available, related studies that utilized allozymes (Chung et al., 2018), microsatellites (Jin et al., 2021), or combinations of DNA sequences with microsatellites and single nucleotide polymorphisms (Cho et al., 2020; Park et al., 2019) as markers only attempted to determine genetic variation and structure, but not to directly infer species demographic history. The knowledge gaps in the demographic history of plant species on the KP, hence, remain understudied specifically on the influence of past events on species' current spatial range and organization.

In this research, we investigated the phylogeographic and demographic historical processes of *Abeliophyllum distichum* Nakai (Oleaceae) to elucidate the relationship between its genetic subdivisions and current fragmented population distribution. More commonly known as white forsythia, this KP endemic and natural monument species is considered rare and is only found in the central provinces of South Korea, although some (unconfirmed) natural occurrences have been also reported from neighboring North Korea (Kim & Maunder, 1998; Son et al., 2016). Despite being sometimes cultivated in its distributional range, the IUCN Red List evaluated the species as endangered due to its severely patchy natural distribution (Son et al., 2016). The most recent genomic survey on the species, which covered its range‐wide distribution, also showed weak or even absence of contemporary gene flow among its populations (Lee et al., 2022).

The species grows on well‐drained substrates on slopes below 200 m asl as an understory shrub in the central region's mixed‐deciduous forests dominated by *Quercus* and *Pinus* species (Chung, 1999; Kim & Kim, 2008; Kim & Maunder, 1998). Its distylous flowers are pollinated by a variety of insects (Chung, 1999; Kang et al., 2000) around late March to early April, while its winged fan‐shaped fruits are wind‐dispersed starting July or August. White forsythia is the sole member in its genus and has general features resembling those of *Forsythia* species, except for its flower color (and fruit type), hence, the common name. *Abeliophyllum* and *Forsythia* are the only genera in the tribe Forsythieae whose divergence was reported to have occurred in East China during the Miocene ca. 5–33.6 million years ago (Ha et al., 2018). Population genetic studies generally agreed that there is moderate to high genetic differentiation in the species (Chung, 1999; Kang et al., 2000). Our recent range‐wide population genomic survey (Lee et al., 2022) supported these initial findings but also discovered patterns of higher genetic variations for populations with central distribution than those found at the periphery.

In this follow‐up study to Lee et al. (2022), we combined the use of single nucleotide polymorphisms (SNPs) to infer the demographic history pattern of the species, and paleodistribution modeling to investigate the species' past suitable habitats. Specifically, our aims were (i) to infer species population divergence/admixture events using approximate Bayesian computation (ABC) modeling and (ii) to reconstruct the species paleoclimatic distribution using ecological niche modeling (ENM). Here, we would like to determine when and how past spatiotemporal events may have influenced the current genetic subdivisions and fragmented range distribution of the species.

## 2 MATERIALS AND METHODS

### 2.1 Sampling, DNA extractions, library preparation, and sequencing

**FIGURE 1:**
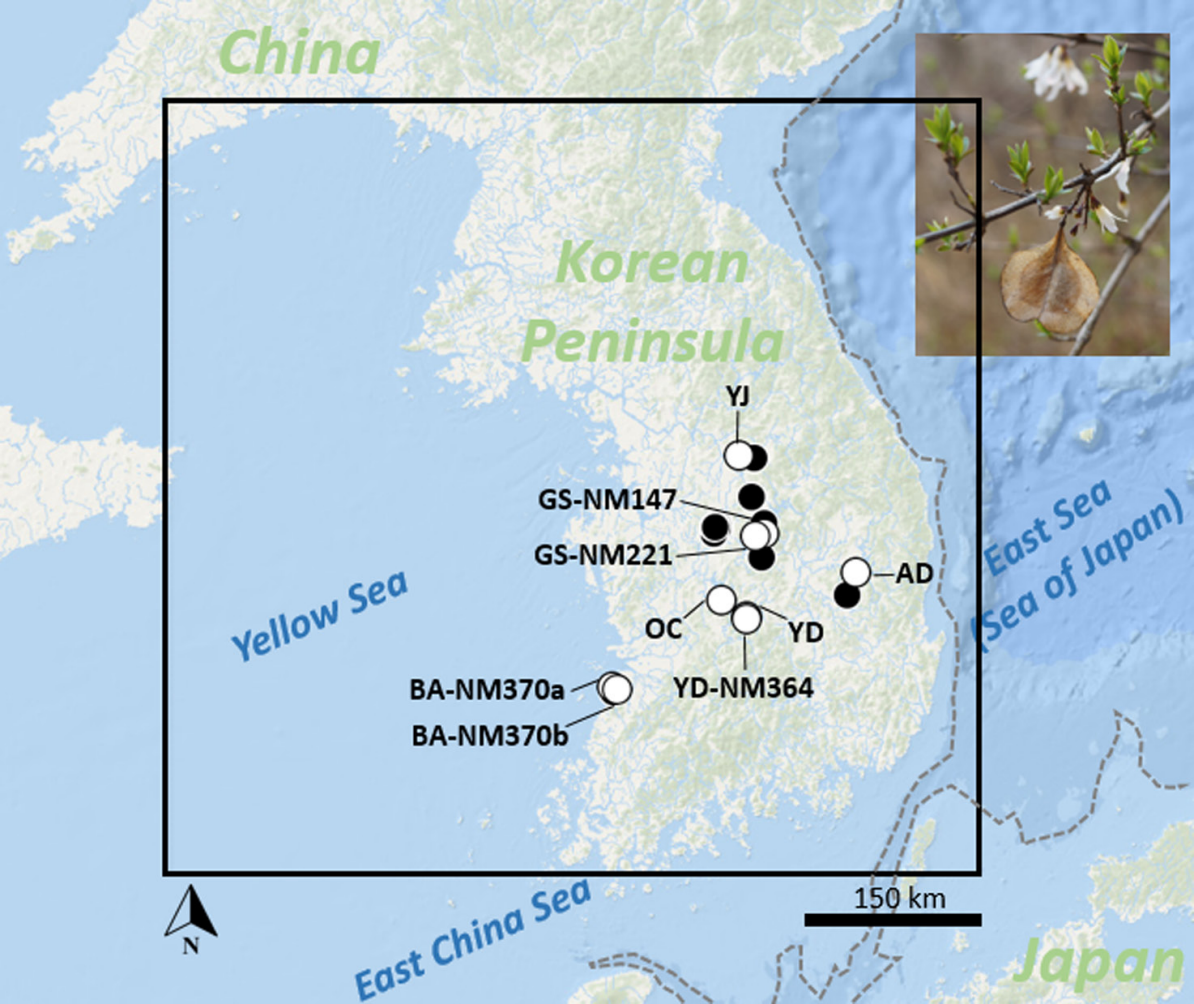
Map showing the natural population distribution of white forsythia (inset) on the KP. White circles (labeled) represent the nine sampled populations used in ABC, while the (unlabeled) black circles represent 13 additional occurrence points (for a total of 22 records) used in ENM. The solid black square marks the calibration area, while the broken gray lines indicate the exposed land during the LGM.

We analyzed 124 white forsythia individuals across nine collection sites (Figure 1), which represented the naturally occurring populations' subsample of our data published earlier (Lee et al., 2022). More detailed information about these sampled locations is shown in Table S1. Genomic DNA from individual samples was obtained from 20 mg of dried leaves/flower buds using a DNeasy Plant Mini Kit following the manufacturer's protocol (Qiagen, Germany). DNA quality was assessed and quantified with Qubit Fluorometer (Invitrogen, USA) and visualized on a 1% agarose gel. For each population, a total of 12–15 samples with the highest DNA quality were selected and then forwarded to Seeders, Inc. (Daejeon, Korea) for GBS library construction (Elshire et al., 2011) and Illumina sequencing. Samples were digested with methylation‐sensitive *ApeKI* restriction enzyme, barcoded with sequence adapters, amplified by polymerase chain reaction (PCR), and sequenced in a single lane on an Illumina HiSeq X (Illumina, USA) using 150 bp paired‐end sequencing runs.

### 2.2 Sequence analyses, bioinformatics, and SNP identification

The sequenced data were demultiplexed using Cutadapt v.1.8.3 (Martin, 2011) and quality‐trimmed using DynamicTrim (phred score ≥ 20) and LengthSort (short read length ≥ 25 bp) as implemented in SolexaQA v.1.13 (Cox et al., 2010). Cleaned reads were mapped against the assembled reference draft genome (see Lee et al., 2022 for draft genome access information). Genetic mapping was done by using the program BWA 0.6.1‐r104 (Li & Durbin, 2009). To detect raw SNPs (In/Del) and extract the consensus sequences, SAM file format was merged and sorted into a BAM using the program SAMtools v.0.1.16 (Li et al., 2009). See Lee et al. (2022) for precise parameter settings.

The final loci matrix was produced by calling only biallelic SNPs, filtering individual missing rate of <30%, and selecting loci with a minor allele frequency (MAF) of >5%. Each locus was tested for Hardy–Weinberg (H‐W) equilibrium (*p* ≤ .01) and *F*_ST_ (>0.1) (Weir & Cockerham, 1984) using the R package *pegas* (Paradis, 2010). Loci which showed high linkage disequilibrium (LD) (*r* ^2^ = 0.8) were filtered out using the software Plink v1.07 (Purcell et al., 2007).

### 2.3 Genetic clustering using multivariate, Bayesian, and phylogenetic methods

Genetic structuring of samples was initially explored using the R package *adegenet* (Jombart & Ahmed, 2011) function called the discriminant analysis of principal components (DAPC), which is a multivariate method that maximizes genetic differentiation between groups. The find. clusters() command was run to determine the number of genetic clusters (*K*) by retaining 120 principal components (PCs). The optimal *K* was determined by selecting the Bayesian Information Criterion (BIC) with the lowest value. Before the final DAPCrun, we selected the optimal number of PCs to use by running the optim.a.score() command and using all 120 PCs and 100 discriminant functions (DAs) to maximize information content. All the above analyses were done in R (R Core Team, 2020).

The program STRUCTURE v2.3.4 (Pritchard et al., 2000) was used to further evaluate population structuring by assigning individuals into genetic clusters based on Bayesian assignment analysis. After setting the prior for the most likely *K* from 1 to 9, 10 independent runs for each value of *K* were performed using a Markov Chain Monte Carlo (MCMC) length of 100,000 generations following a burn‐in of 50,000 generations. We selected the admixture and correlated allele frequencies options and set all other parameters to default. We calculated the ΔK (Evanno et al., 2005) to determine the optimal number of genetic clusters using Structure Harvester (Earl & VonHoldt, 2012).

To elucidate the phylogenetic relationship among the sampled populations, we constructed a coalescence‐based tree using SNAPP (Bryant et al., 2012), with input data prepared using BEAUTI, as implemented in BEAST v.2.0 (Bouckaert et al., 2014, 2019). This method estimates species trees from biallelic markers but bypasses the necessity of having to explicitly sample the gene trees at each locus (Bryant et al., 2012; Leaché & Bouckaert, 2018). Due to the high computational demand, we ran the program using a random sample subset consisting of 18 individuals across the nine sampled populations (i.e., two accessions per population). We calculated the forward (*u*) and reverse (v) mutation rates by selecting the “Calc mutation rates” option and set the MCMC algorithm to 1 million generations and a burn‐in of 200,000 generations. Trees were sampled every 1000 steps, while other options were set to default. The coalescence tree was further processed and visualized using TreeAnnotator (Bouckaert et al., 2014, 2019) and FigTree v1.4.3 (http://tree.bio.ed.ac.uk/software/figtree/), respectively. The resulting tree topology, along with the results of population structure analyses, was referred to in designing demographic history models to be tested using ABC.

### 2.4 Demographic history analysis using ABC

To look into the demographic history of white forsythia, we employed ABC, a powerful and flexible approach for estimating demographic and historical parameters by testing and comparing the most probable evolutionary and demographic history models (also known as alternative scenarios; Bertorelle et al., 2010). The ABC framework attempts to obtain the posterior distribution of the parameters by simulating genetic datasets under a given demographic model (Beaumont, 2010; Beaumont et al., 2002). We used the ABC framework implemented in DIYABC v2.1, which calculates the estimated time of divergence and/or admixture of genetic groups, as well as their effective population size distributions by evaluating the summary statistics (sumstats) and predefined priors of the evolutionary and demographic history models (Cornuet et al., 2014).

To simplify the analysis, the nine sampled populations were pooled into five groups based on the results of genetic clustering and phylogenetic analyses (see Figure 4 in Results). To further reduce the computational load and increase the accuracy of estimates, we only selected SNP loci that contained no missing data. The input file was prepared using Hudson's simulation algorithm for SNP markers (Hudson, 2002), which is equivalent to setting the MAF criterion of the program to default (Cornuet et al., 2014).

**FIGURE 2:**
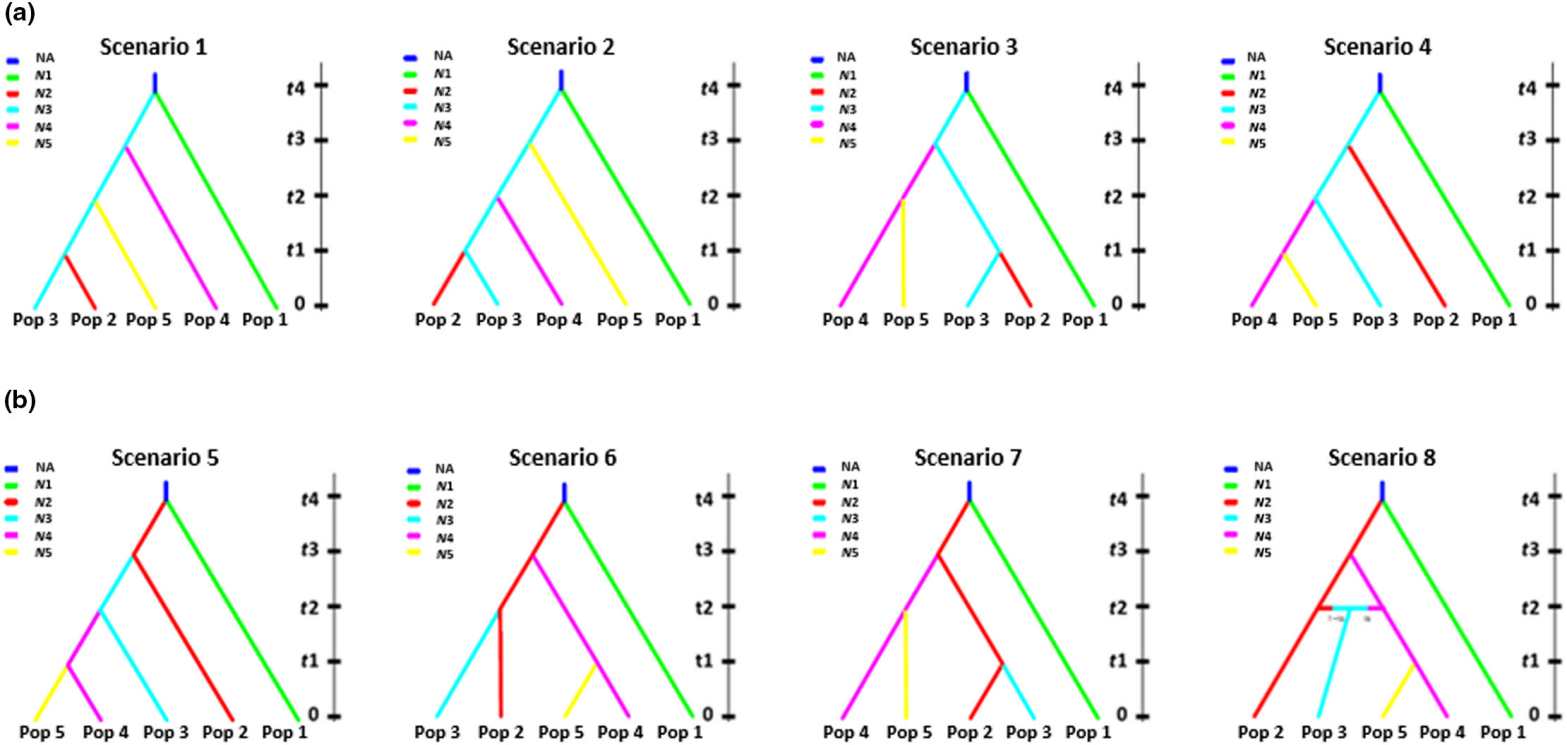
Eight demographic scenarios are categorized into two main groupings: (a) UPPER‐CENTRAL Divergence Models (Scenarios 1–4) and (b) LOWER‐CENTRAL Divergence Models (Scenarios 5–8), suggesting the demographic history of events of the species' five genetic subdivisions. See Note S1 for detailed description and model assumptions. *N*1/Pop 1 = SOUTHERN, *N*2/Pop 2 = LOWER‐CENTRAL, *N*3/Pop 3 = UPPER‐CENTRAL, *N*4/Pop 4 = EASTERN, *N*5/Pop 5 = NORTHERN, NA = ANCESTRAL POPULATION.

We designed our models based on our earlier findings on white forsythia distribution, specifically by following the signals supporting the central‐marginal hypothesis (Lee et al., 2022). After several preliminary runs on a larger (i.e., 11 total) number of alternative scenarios using different combinations of sumstats and prior estimates, we ended up with eight final models to test, as shown in Figure 2 (see also Note S1 for each model description and scenario assumptions). We set the uniform priors from 10 to 6 × 10^5^ for the estimation of the effective population size (*N*), 10 to 10^3^ for the estimation of divergence times (*t*) with *t*1 < *t*2 < *t*3 < *t*4, and 10^−3^ to 0.999 for admixture rate (ra) computation. See Table S2 for specific prior parameter settings.

Overall, we ended up with 11 parameters and 26 sumstats combinations to generate a reference table based on 8 × 10^6^ simulated datasets (ca. 1 million runs for each scenario). The selected sumstats were as follows: mean of complete distribution for single sample statistics (i.e., mean gene diversity (Nei, 1987) across all loci), mean of non‐zero values for two sample statistics (i.e., mean *F*_ST_ distances (Weir & Cockerham, 1984), and mean Nei's distances (Nei, 1972) across loci with non‐zero values between two samples), and mean admixture estimates across loci with non‐zero values for three‐sample statistics (Choisy et al., 2004).

To determine the most highly supported scenario(s) among the eight candidate models, posterior probabilities (PPs) were computed via the direct approach based on 500 datasets, and the logistic regression based on the 1% of simulated datasets closest to the observed data after replacing the original sumstats by discriminant scores (Cornuet et al., 2014; Estoup et al., 2012). We then calculated the confidence among the top scenario candidates by computing the scenario‐specific prior‐based error (also known as the prior error rate). Here, 1000 pseudo‐observed datasets were drawn from parameter prior distribution under the best‐fit scenario by choosing the direct approach (based on the 500 closest datasets), and the logistic regression (after selecting 1% of simulated datasets) for each of the top competing scenario candidates (Cornuet et al., 2014). The best demographic history model was chosen by calculating the number of times the most highly supported scenario did not have the highest PP when it was the true scenario (i.e., type I error/false positive).

To evaluate how well the most highly supported scenario and its prior and posterior parameters fit the data (i.e., the goodness‐of‐fit of the model), we ran the DIYABC model checking option using the above‐described (applicable) sumstats. Another set of sumstats was also used for a second, less biased model checking, a method that is expected to reduce the risk of overestimating the quality of the fit by avoiding the use of the same sumstats (Cornuet et al., 2010). Finally, using the DIYABC parameter estimation, the posterior distribution parameters (e.g., *t* and *N*) for the scenario of choice were computed by selecting the logit transformation on the 1% of the closest simulated datasets. The mean posterior distribution for *t* was then recalibrated to the absolute divergence time (i.e., into years) by multiplying it with the generation time of the species, which was set to 8 (6–10 years in Lee et al., 2022).

### 2.5 Present and past habitat suitability analyses using ENM

White forsythia occurrence points used in ENM (also called species distribution modeling), were taken from our sampled sites and herbarium collections deposited in major herbaria in South Korea (KH, KB), and the Herbarium of Hallym University (HHU). To control the quality of occurrence data, we removed duplicate records, limited our collections from sites where the species only naturally occurs, and filtered them at ca. 1 km radius using the *spThin* package (Aiello‐Lammens et al., 2015) in R (R Core Team, 2020). Note that reports of occurrence(s) from North Korea could not be validated via voucher specimens, hence, were not included in the selection process. Overall, 22 occurrence points (see Table S1) were retrieved for the subsequent ENM‐related preparations below.

To find potentially suitable habitats from the present and project them to the past, we delimited our study region to a relatively larger calibration area that covered the geographic extent of the KP (see Figure 1). We acknowledge that the study region occupies areas that the species may not be able to disperse (e.g., present seas and coasts), thus, our output maps should be taken with caution. We downloaded a total of 19 current (1970–2000) bioclimatic variables (e.g., temperature and precipitation predictors) from WorldClim database v1.4 (Fick & Hijmans, 2017). The variables represented the highest spatial resolution of 30 arcsec or ca. 1 × 1 km and comprised sumstats at different temporal resolutions interpolated from WorldClim weather station data. The geographic information system software QGIS v3.22.5 (QGIS Development Team, 2020) was used to retrieve the environmental layers of the study region. Spatial resolutions of the different environmental layers were uniformly adjusted using the R package *raster* (Hijmans et al., 2015).

To determine the most biologically relevant environmental variables for the species, we tested a set of candidate distribution models. Using the R packages *ENMTools* (Warren et al., 2021) and *usdm* (Naimi, 2015), we excluded the models that showed combinations of highly correlated variables (*r* ≥ 0.7) and considered the removal of variables with very high variance inflation factor or VIF (>10). We also conducted an initial screening of all 19 environmental variables in MAXENT v3.4.4 (Phillips et al., 2017) based on a stepwise removal using the jackknife test and excluded the ones with the least percent contribution (<1%) to the overall model. The environmental predictors that showed the highest contribution to the model prediction are the following: maximum temperature of the warmest month (bio5), annual range of air temperature (bio7), annual precipitation amount (bio12), precipitation amount of the wettest month (bio13), and precipitation amount of the driest month (bio14). The above final five environmental layers were selected for all the subsequent MAXENT runs.

Before the final runs, we optimized candidate niche models using the R package *ENMeval* (Muscarella et al., 2014) to avoid model over‐prediction. Over‐fitting and complexity of the model were evaluated by varying the regularization multiplier (RM) (e.g., 0.5, 1, 1.5, 2, 2.5, 3, 3.5, 4) and by making different combinations of the following constraints or feature classes: linear (L), quadratic (Q), hinge (H), and product (P) (e.g., L, Q, H, P, LQ, LH, LP, QH, QP, HP, LQH, QHP, LQHP). We selected the jackknife partition settings to 22 (corresponding to the 22 occurrence points), resulting in a total of 104 niche model candidates and the production of a sampling bias file. The best niche model parameters were selected by choosing the delta.AICc with the lowest value (i.e., zero) for the final maximum entropy run.

In MAXENT v3.4.4, we selected the complementary log–log (cloglog) output to estimate the probability of presence, as recommended by Phillips et al. (2017). Our *ENMeval* optimization gave the following best parameter combinations: RM of 1.5, LH feature classes, and a maximum number of background points of 10,000. Additionally, the following options were selected: initial random seed for each iteration, jackknife to measure variable importance, crossvalidation as a method of replication by running 22 replicates, a maximum iteration of 1000, default prevalence of 0.5, the use of sampling bias file, and the threshold rule of 10% training presence. All other parameters were left in their default settings. To assess the discriminatory capacity of the predicted niche model, we referred to the area under the curve (AUC) of the receiver operating characteristic (ROC). AUC values determine the relative suitability of habitats with values ranging from 0 to 1.

The model that was calibrated onto the Present (1970–2000) conditions was projected to the past climatic conditions using the same five environmental predictors downloaded from CHELSA‐TraCE21k v1.0, which provides high‐resolution climatic data from 21 kya to 1990 in 100‐year time steps (Karger, Conrod, et al., 2021, Karger, Nobis, et al., 2021). In this paper, we decided to model the species' paleodistribution in the LGM (21 kya), Early Holocene (10 kya), Mid‐Holocene (6 kya), and Late Holocene (3 kya). The resulting continuous map models were then converted to binary output maps to visualize the suitable and unsuitable areas (or presence and absence of distribution) from the present to the different times in the past, following the threshold value for each temporal model. Visualization of ENM maps was prepared using QGIS v3.22.5 (QGIS Development Team, 2020).

## 3 RESULTS

### 3.1 Sequence analyses, bioinformatics, and SNP identification

The Illumina sequencing on our *ApeKI* GBS library produced an average of ca. 600 million trimmed reads per sample and ca. 500,000 SNP loci matrix. After checking for sequence quality and minimum read depth, we were able to recover ca. 27,000 loci, each containing a single biallelic SNP, with an average read depth of 25X per marker. Quality control for coverage across individuals, MAF, and further filtering steps of H‐W equilibrium, LD, and *F*_ST_ identified a total of 1897 SNP loci. This set of loci was used for the characterization of genetic structure and phylogenetic analysis, while another set of 693 SNPs, which are loci with all missing data removed, was used for ABC demographic history analysis.

### 3.2 Genetic clustering using multivariate, Bayesian, and phylogenetic methods

**FIGURE 3:**
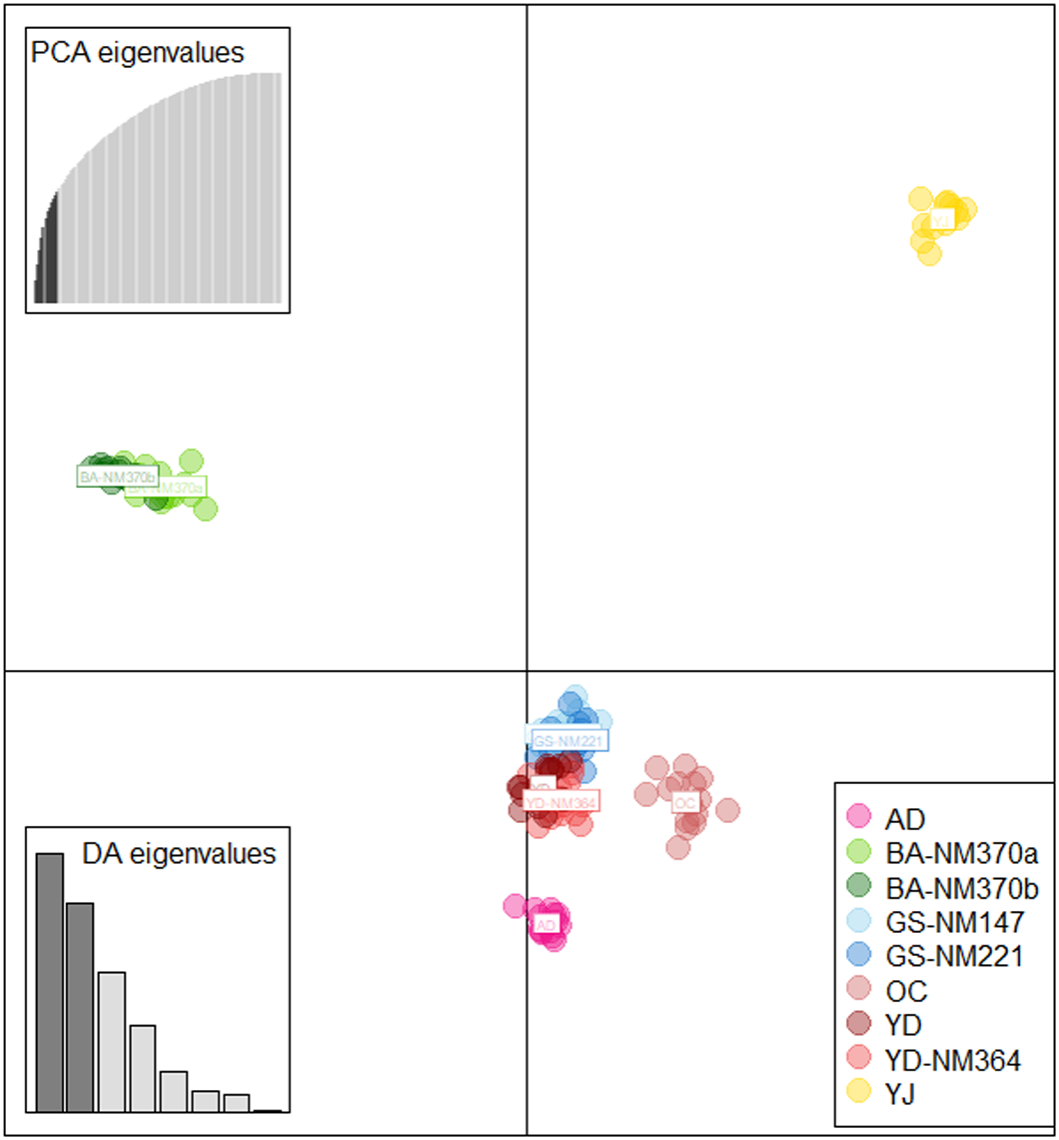
DAPC plot showing the clustering of 124 individuals across nine sampled populations on the first two axes of the 11 retained PCs (upper left inset) and eight DAs (lower left inset).

The DAPC analysis, even with groups undefined, showed an optimal K of 9 and suggested retaining 11 optimal numbers of PCs after the optim.a.score() command (Appendix 1). The final DAPC plot (Figure 3) shows the clustering of samples on the first two axes of the 11 retained PCs and eight DAs. In axis 1, genotypes from the southernmost populations BA‐NM370a and BA‐NM370b are shown positioned to the far left, and thus, clustered very distinctly from other populations. In axis 2, the northernmost population YJ and the easternmost population AD are respectively plotted relatively farther above and below the populations that comprised the core central cluster. The core central cluster (GS‐NM147, GS‐NM221, OC, YD‐NM364, and YD) appears to be very close and almost indiscernibly grouped.

**FIGURE 4:**
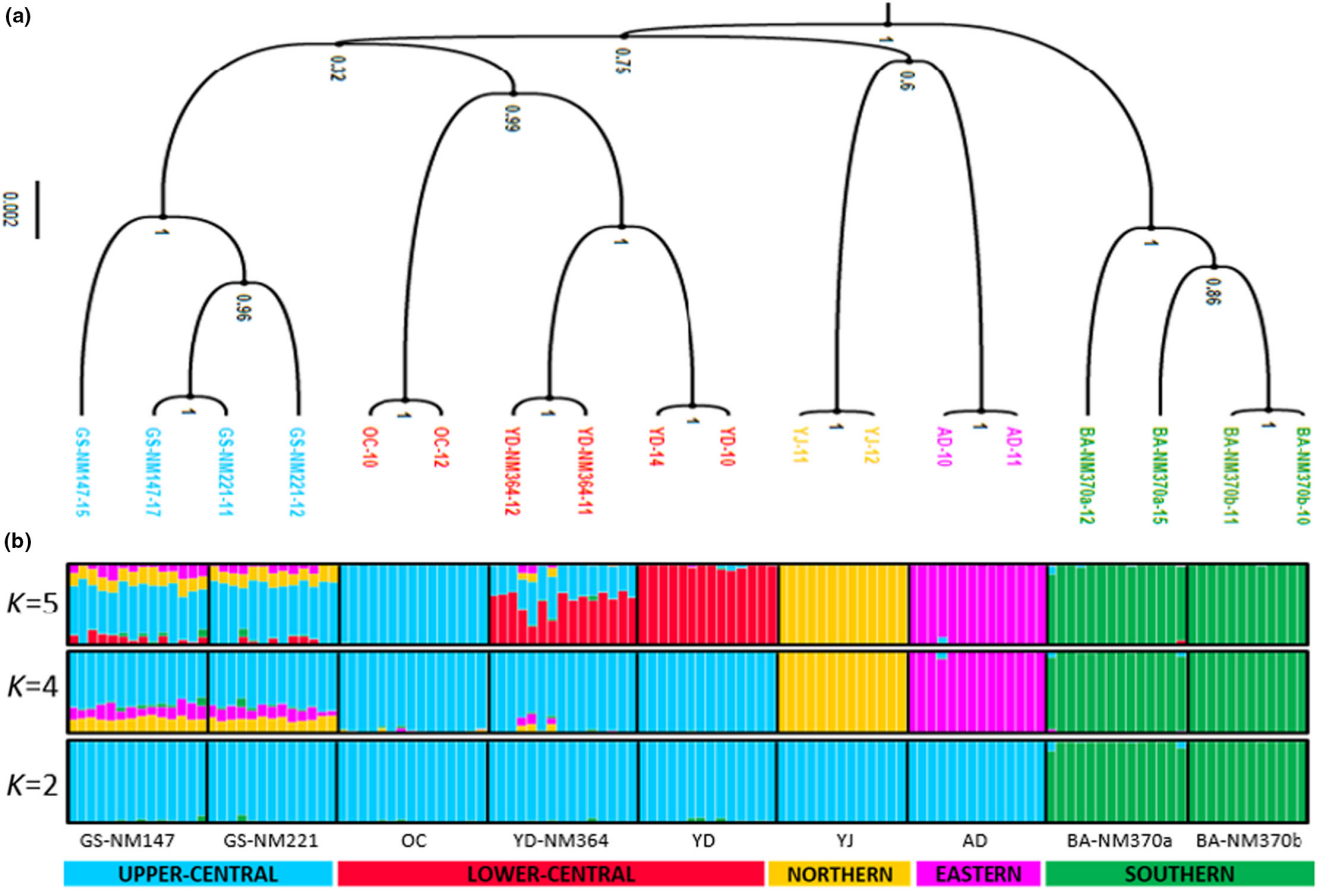
(a) SNAPP coalescent tree topology of 18 random samples (two per population). Posterior probability values are shown at nodes. (b) Results of STRUCTURE analysis, showing the optimal number of clusters (*K* = 2, *K* = 4, and *K* = 5) inferred from 124 samples as represented by individual bars, and their assignment across the nine sampled populations as delineated by solid black lines.

A more delineated genetic grouping can be visualized when samples are classified into optimal *K*s in STRUCTURE analysis (Figure 4b). Δ*K* estimates reveal *K* = 2, *K* = 4, and *K* = 5 as the most probable number of genetic groups (Appendix 2). Figure 4b shows that at *K* = 2, two initial lineages are formed and composed of the two southernmost populations BA‐NM370a and BA‐NM370b (green genetic assignment), which remained genetically distinct as K increased both at *K* = 4 and *K* = 5, and all the remaining populations (blue genetic assignment). At *K* = 4, the northernmost population YJ and the easternmost population AD are shown to have diverged from the “blue” genetically assigned cluster, which now consisted only of populations from the core central range. At *K* = 5, this core central cluster can be seen divided into a lower‐central geographic group comprising OC, YD‐NM364, and YD, and an upper‐central geographic group composed of GS‐NM147 and GS‐NM221. While members of the upper‐central group maintained a fairly uniform genetic identity until *K* = 5, the genetic assignment of populations into the lower‐central group appears to be due to admixture (i.e., YD‐NM364 as a product of OC and YD parental populations) and is attributable to their geographic proximity.

Finally, our SNAPP analysis infers *K* = 5 as the most probable number of genetic clusters (Figure 4a). The coalescent tree topology, generally with strong PP support, reveals that randomly selected samples per population formed clades based on their (phylo)genetic relationship and sampled locations. The tree suggests that after divergence from a putative ancestral population, two main lineages (PP = 1.00) are formed: the clade of the southernmost group (BANM370a and BA‐NM370b), which appears to be the most basal (PP = 1.00) and that of all remaining populations (PP = 0.75). Within the latter clade, the easternmost AD and the northernmost YJ are shown to closely cluster (PP = 0.60), distinct from the clade of the populations with core central geographic distribution (PP = 0.32). Note that albeit unresolved, the topology suggests the close relationship between the two groups with central distribution: GS‐NM147 and GS‐NM221 populations for the upper‐central geographic group (PP = 1.00), and the OC, YD, and YD‐NM364 populations for the lower‐central geographic group (PP = 0.99). Based on these findings, we finalized the following a priori genetic units: SOUTHERN, NORTHERN, EASTERN, UPPER‐CENTRAL, and LOWER‐CENTRAL groups. All subsequent ABC model testing and analyses were based on these genetic units. See Table S1 for complete information about the sampled populations in each grouping.

### 3.3 Demographic history inferred by ABC models

Among the eight alternative scenarios, our ABC analysis identified Scenario 1 and Scenario 2 as the top two competing models, as shown in Table 1 (see also Appendix 3 for the graphical visualization of model comparison and selection). PP estimates with 95% confidence intervals (CI) reveal that under the logistic regression approach, Scenario 1 showed a higher estimate (PP = 0.525, CI = 0.243–0.862) than Scenario 2 (PP = 0.429, CI = 0.061–0.996), while under the direct approach, the former revealed a lower estimate (PP = 0.183, CI = 0.000–0.522) than the latter (PP = 0.473, CI = 0.042–0.909). The scenario‐specific prior‐based error, however, indicates that the scenario of choice is Scenario 1, as shown by the type I error on both the logistic regression (92.3%) and the direct approach (87.5%) (Table S3).

**TABLE 1:** Eight demographic history scenarios (top two competing models in bold), and their posterior probability estimates and 95% confidence intervals (in brackets).

Scenario	Direct	Logistic regression
**1**	0.18319 [0.0000–0.5220]	0.52499 [0.2429–0.8620]
**2**	0.47297 [0.0417–0.9092]	0.42874 [0.0613–0.9963]
3	0.09748 [0.0000–0.3570]	0.00928 [0.0000–0.5470]
4	0.04069 [0.0000–0.2114]	0.00008 [0.0000–0.5442]
5	0.03505 [0.0000–0.1961]	0.00147 [0.0000–0.5446]
6	0.03651 [0.0000–0.1976]	0.00258 [0.0000–0.5448]
7	0.12493 [0.0000–0.4142]	0.03220 [0.0000–0.5733]
8	0.00916 [0.0000–0.0835]	0.00066 [0.0000–0.5444]

**FIGURE 5:**
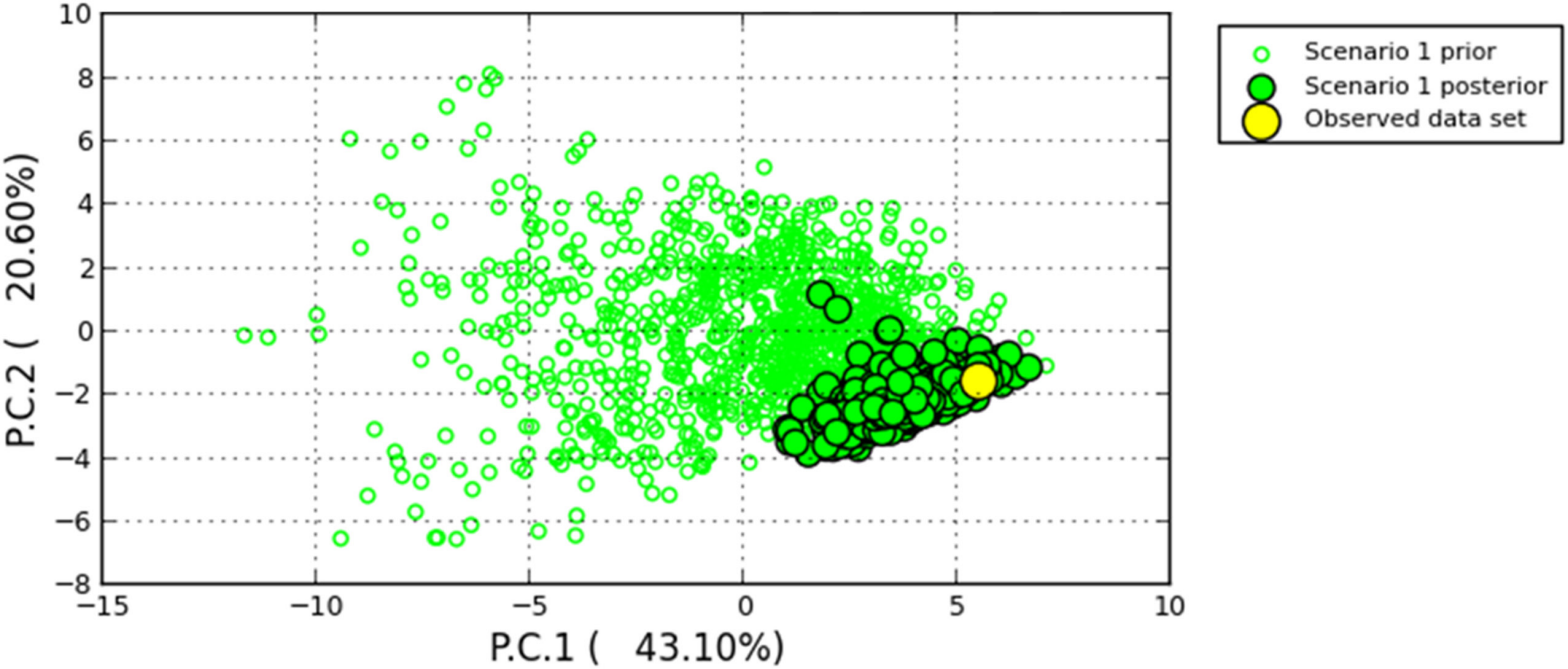
PCA plot of the goodness of fit of the most highly supported model (i.e., Scenario 1) as shown by the observed dataset (yellow circle) being nested within the posterior distribution (black‐outlined green circles). Hollow (green) circles indicate the spread of the prior distribution.

The goodness‐of‐fit of Scenario 1 and its parameters were further confirmed by checking the position of the observed dataset in the space of sumstats on a PCA plot (Figure 5). The observed dataset is shown nested within the posterior distribution, as explained by 43.1% and 20.6% proportions of variance in PC1 and PC2, respectively. Even when a less biased goodness‐of‐fit computation was conducted (using a different combination of unused sumstats), Appendix 4 does not show the observed dataset being plotted outside the posterior distribution.

Table 2 parameter estimates for Scenario 1 present the posterior distribution of the mean effective population size (*N*) for each genetic group, as well as that of the mean divergence times (*t*) during each event. After considering the generation time of 8 (6–10) years, it can be interpreted that the ANCESTRAL POPULATION (NA = 598,000) was split into a basal SOUTHERN group (*N*1 = 3440) and a large central metapopulation lineage (i.e., that of the UPPER‐CENTRAL group) at *t*4 ca. 10 kya. All the remaining populations then subsequently diverged from the latter lineage. For instance, the EASTERN group (*N*4 = 681) and the NORTHERN group (*N*5 = 365) were formed from this lineage after their split at *t*3 ca. 5 kya and at *t*2 ca. 3 kya, respectively. Lastly, the split of the core central lineage into the LOWER‐CENTRAL group (*N*2 = 2710) and the UPPER‐CENTRAL group (*N*3 = 2600) appears to have only occurred at *t*1 ca. 2 kya. See Appendix 5 for Scenario 1 graphical parameter estimates of both the prior and posterior distributions, and also Table S4 for other parameter estimation statistics (e.g., median, mode, quantiles) of its posterior distribution.

**TABLE 2:** Parameter estimation of the posterior distribution for Scenario 1, showing the mean effective population size (*N*) for each genetic group, and the mean divergence time (*t*) during each event.

Parameter	Genetic group/unit	Mean	Absolute divergence time (in years)
NA	ANCESTRAL POPULATION	5.98E + 05	
*N*1	SOUTHERN	3.44E + 03	
*N*2	LOWER‐CENTRAL	2.71E + 03	
*N*3	UPPER‐CENTRAL	2.60E + 03	
*N*4	EASTERN	6.81E + 02	
*N*5	NORTHERN	3.65E + 02	
*t*4		1.26E + 03	10,080 (7560–12,600)
*t*3		6.29E + 02	5032 (3774–6290)
*t*2		3.51E + 02	2808 (2106–3510)
*t*1		2.61E + 02	2088 (1566–2610)

*Note*: The absolute divergence time (in years) was calculated by multiplying the mean with the generation time of the species set at 8 (6–10) years (Lee et al., 2022).

### 3.4 Past to present habitat suitability inference using ENM

Based on 22 natural occurrence records, five layers of environmental variables, and 22 replicate runs with optimized settings, MAXENT inferred the species' Present (1970–2000) potentially suitable habitats with a predictive power of AUC = 0.878 (SD = 0.125), as displayed in the ROC curve (Appendix 6). This value suggests that the Present (1970–2000) niche model is more accurate to data discriminating than at random (i.e., AUC higher than 0.5), as shown by the curve that is above the diagonal line of no discrimination. An AUC value of 0.75 and above may correspond to high discrimination performances (Fielding & Bell, 1997).

When the five environmental variables were tested, bio14 (46.7%), bio5 (34.5%), and bio7 (15.1%) were revealed to have the highest combined percent contribution to the model (ca. 96% total). The same three variables also showed the highest permutation importance amounting to ca. 97% (Table 3). The response curves for all five variables were shown to be all single‐peaked and more or less normally distributed (except for bio13), with those for bio14 driest month precipitation amount indicating the highest suitability peaking at ca. 24 mm, bio5 warmest month temperature of ca. 29°C, and bio7 annual range of air temperature of ca. 38°C (Appendix 7).

**TABLE 3:** Five environmental variables and their contribution and importance to the Present (1970–2000) niche model.

Variable	Variable name and unit ()	Percent contribution	Permutation importance
bio14	precipitation amount of the driest month (mm)	46.7	48
bio5	maximum temperature of the warmest month (°C)	34.5	32.2
bio7	annual range of air temperature (°C)	15.1	17.1
bio13	precipitation amount of the wettest month (mm)	3.6	2.1
bio12	annual precipitation amount (mm)	0.2	0.6

After the Present (1970–2000) habitat suitability was projected onto the four paleoclimatic and geographic conditions, binary output maps chronologically revealed a pattern of directional shifting and size changes in the species' past suitable habitats (Appendix 8). Among these temporal models, the LGM (21 kya) displays the smallest suitable area with the greatest density located west of the central KP. The Early Holocene (10 kya) model that followed shows an obvious increase in habitat suitability, which appears to have shifted and expanded eastward and northward to the center of the KP. By the Mid‐Holocene (6 kya), and Late Holocene (3 kya), a somewhat subsequent decrease in habitat suitability is displayed. Finally, the Present (1970–2000) niche model depicts the most continuous suitable conditions for all species population occurrences.

## 4 DISCUSSION

### 4.1 Population fragmentation after sequential divergence events

**FIGURE 6:**
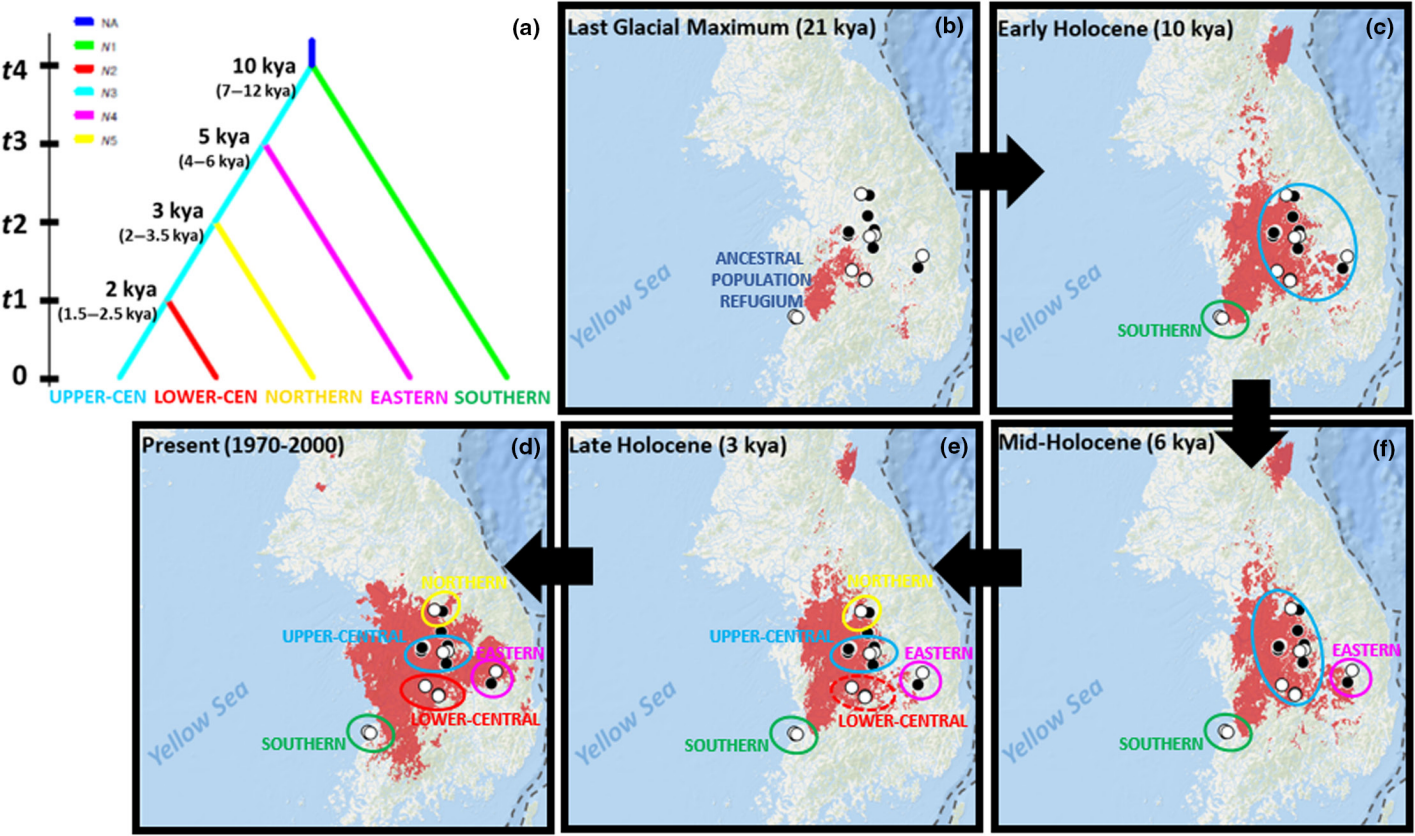
Comparison of ABC (a) and ENM (b–f) results. (a) Tree topology of the most highly supported model (Scenario 1) showing the absolute divergence time of events (in kya) at nodes and computed from the generation time of the species which was set at 8 (6–10) years. (b–f) ENM of the species' suitable habitats (red) from past to present (indicated by arrows), suggesting the direction of divergence/expansion events that formed the five (hypothetically inclusive) genetic subdivisions in white forsythia (encircled and labeled). White and black circles represent the sampled sites and additional occurrence points, respectively.

Based on the most highly supported ABC model (i.e., Scenario 1), the demographic history of white forsythia was inferred to be a post‐LGM sequential population divergence of the EASTERN, NORTHERN, and LOWER‐CENTRAL genetic groups from the UPPER‐CENTRAL lineage, following the latter's split with the SOUTHERN group that commenced at the onset of the Holocene (Figure 6a). Although our molecular data were not able to show any demographic events (e.g., divergence, admixture) during the last glacial period, our ENM (Figure 6b) was able to infer the existence of a suitable habitat west of the KP during the LGM (ca. 21 kya). The suitable habitat appeared to have been situated between the species' current southernmost and central ranges and could represent a past glacial refugium, albeit restricted to a reduced area. Although this refugium may appear to have been nearly coastal in distribution relative to the present time, during the LGM, the sea levels in East Asia were so low that the continental shelves of the Yellow Sea (and the East China Sea) became sub‐aerially exposed (Bloom & Park, 1985; Kong et al., 2006; Park et al., 1994). Clark et al. (2019) reported that this spatiotemporal event may have allowed the survival and even dispersal of widespread grass species of *Miscanthus* across the region. In our LGM model (Figure 6b), however, no white forsythia suitable habitat is projected in the exposed Yellow Sea, a similar finding even to a more commonly distributed, related species *Forsythia suspensa* (Fu et al., 2014). This means that during the LGM, the exposed portion of the Yellow Sea, or at least the calibrated area covered in our analysis, may not have provided a hospitable environment for this taxonomic group. On the contrary, palynological evidence from western central KP revealed that during this glacial period (ca. 22.5–20.5 kya), conifers and cool temperate deciduous broad‐leaved mixed forests predominated the mountainous areas under relatively cool and wet conditions, before transitioning to a later drier and colder period (ca. 20 kya) when subalpine coniferous forests reoccupied the montane regions and hinterlands (Yi & Kim, 2010). The prevailing climates during the period (ca. 26–21 kya) were reported to be 5–6°C colder and drier than at present (Kim et al., 2015; Yi & Kim, 2010).

By Early Holocene (ca. 10 kya), Figure 6c reveals the expansion of the glacial refugium slightly southward, giving way to the establishment of the SOUTHERN group (green cluster), and more predominantly inland, forming the central metapopulation lineage (large blue cluster) that would later divide into and/or migrate to form other genetic subdivisions. Our ABC model (Figure 6a) shows that the first demographic event occurred at *t*4 ca. 10 (7–12) kya when the SOUTHERN group and the larger central metapopulation lineage (i.e., that of the UPPER‐CENTRAL group) diverged from the ANCESTRAL POPULATION NA. The north‐eastward expansion of suitable habitats was most likely due to the submergence of the western side of the KP during deglaciation events. Severe post‐glacial sea‐level rise, which started ca. 18–19 kya, resulted in rapid marine transgression of the exposed Yellow Sea continental shelf due to high tidal effects and the low seafloor gradient (Kong et al., 2006; Yoo et al., 2016). This marine transgression continued and reached its maximum in the Mid‐Holocene, when seawater invaded farther inland from the west coast, even leading to bay environments in the alluvial plains downstream of major rivers in central Korea (Yoon et al., 2012). Despite projections of suitable conditions (based on five climatic predictors) on some portion of the westernmost region, soil salinity, a variable not accounted for in this study, may have prevented the possible distribution of white forsythia near the western coast, except the SOUTHERN group, which is currently the only extant population closest to the Yellow Sea. This emphasizes the identity of this basal lineage as a stable, rear‐edge population (Hampe & Petit, 2005) which remained unmixed and has been in existence in situ since before the Early Holocene, not to mention the conservation significance of the protected area (Byeonsanbando National Park) where this genetic group is found (Leem et al., 2020).

Although the increased sea level may have limited the species’ distribution on the western side of the peninsula during the Early Holocene, the geological events and warmer climates may also have paved the way to the extension of preferable environments inward the KP. Around this period (ca. 9 kya), according to Kutzbach (1981), the strengthened solar radiation, which was ca. 7% greater than at present, caused an increased heat difference between land surfaces and the surrounding oceans, giving rise to more intensified East Asian monsoons. The expansion of white forsythia colonies may have been influenced by increased precipitation associated with the monsoons or by the stronger summer (and winter) winds that promoted fruit dispersal further inland. The combination of the strengthened East Asian monsoons and the submergence of the continental shelves did not only carry an increase in humidity but also in temperature (Chung et al., 2006), and likely affected the spread of vegetation where white forsythia may have co‐occurred. According to Yoon et al. (2012), as post‐glacial warming began in the KP (ca. 7–10 kya), *Alnus*‐ and *Quercus*‐dominated forests prevailed in the western regions, suggesting a wetter environment in the area, while the prevalence of *Pinus* forests in the east still implied a relatively drier and cooler eastern region.

By Mid‐Holocene (6 kya), only a very slight reduction in areas of suitability is shown (Figure 6f). Small patches of preferable environments north and south of the main cluster appear to have been wiped out, but those found between the EASTERN group (pink cluster) and the remains of the large central metapopulation (blue cluster) appear to have persisted (Figure 6f). This model corroborates with the findings in our ABC model (Figure 6a), supporting the former's divergence from the latter at *t*3 ca. 5 (4–6) kya, most likely via migration. During this time (ca. 5–7 kya), according to Yi et al. (2008), the East Asian monsoon remained strong, reflecting Mid‐Holocene hypsithermal (i.e., warmest post‐glacial climatic) conditions. Temperatures during the Mid‐Holocene hypsithermal optimum rose to as much as 1–4°C above present temperatures (Kim et al., 2014). Recovered pollen records from central KP suggested that the enhanced climatic events promoted the optimal expansion of a variety of vegetation, such as deciduous broad‐leaved and evergreen mixed forests (Yi et al., 2008). The further eastward expansion of white forsythia, however, may have been held back by the rugged mountain ranges located on the eastern region of the peninsula.

By Late Holocene (ca. 3 kya), potential paleodistribution further decreased but suitable environments between the core central population and the NORTHERN group (yellow cluster) appears to have been maintained, as shown in Figure 6e. This implies that in our ABC analysis (Figure 6a), the formation of the NORTHERN group at *t*2 ca. 3 (2–3.5) kya was likely a result of poleward migration of the core central lineage. It is important to note, however, that there is a narrow chronological margin between *t*2 and the preceding *t*3 (i.e., ca. 5 (4–6) kya). This discrepancy may have resulted in the tight selection between the top two competing demographic models and on whether eastern divergence occurred first followed by northern divergence after migration (i.e., Scenario 1), or otherwise (i.e., Scenario 2). The further poleward migration of the NORTHERN group during the Late Holocene, however, may have been hindered by the hilly geography approaching north, and/or by the cooler temperatures that came along with the increase in elevation and latitude. We also think that the eventual separation from the core central population of the NORTHERN group (and other potentially expanding populations) may have been associated with another major climatic transition: the shift from Mid‐Holocene hypsithermal conditions to a cooler and drier Late Holocene climate. This climatic change is supported by the evidence of the abrupt decline of pollen from *Alnus*‐ and *Quercus*‐dominated forests and the increased amount of pollen from *Pinus* and other cool‐adapted taxa like *Picea*, *Abies*, and *Betula* in the region (Yi et al., 2008; Yoon et al., 2012).

Finally, our ABC model shows that the most recent divergence event in white forsythia occurred at *t*1 ca. 2 (1.5–2.5) kya, delineating the UPPER‐CENTRAL and LOWER‐CENTRAL groups (Figure 6a). The Late Holocene divergence supports an earlier hypothesis that these subpopulations may have been once part of a larger, continuous, or at least less structured core central population before their eventual fragmentation (Lee et al., 2022). Our coalescent tree (Figure 4a), however, gave weak support to this split, probably either due to the admixed assignment of samples in the group or the recency of the event. This split into two sub‐central groups, despite the continued presence of suitable habitats between them (Figure 6e), implied population divergence due to anthropogenic forces. Forests in central Korea have been primarily affected by agriculture and human disturbance since 2 kya (Choi, 1998; Yi et al., 2005) or even earlier (see Lee, 2011; Kwak et al., 2017 for evidence of agricultural practices), as indicated by the decline in pollen of deciduous broad‐leaved trees and the increase of pollen from secondary conifer forests, cultivated grasses (Poaceae), and buckwheat (*Fagopyrum*) (Yi et al., 2008). The considerable impacts of anthropogenic activity on regional climate, however, were said to have commenced at ca. 1.3 kya and became more pronounced 425 years ago (Song et al., 2018). Thus, we believe that despite the generally continuous suitable Present (1970–2000) environments (Figure 6d), human‐induced factors may have made the (already) climate‐driven discontinuous populations of white forsythia more severely fragmented into the habitat islands that we see today.

### 4.2 Research highlights, limitations, and recommendations

In this study, we determined the processes driving the phylogeographic structure of white forsythia by combining the use of ABC on genome‐wide SNP data and ENM reconstruction of paleoclimatic habitats to infer a more accurate demographic history of the species. Our work is a follow‐up to a genomic survey on the species' range‐wide populations (Lee et al., 2022) wherein genetic diversity values in the central ranges were observed to be higher than those at the periphery (i.e., central‐marginal hypothesis). This finding motivated us to seek answers to how these genetically structured populations may have diverged. In the present study, we aimed to determine the directions of these divergence events (i.e., from which “abundant center” to which less diverse margins), as well as to infer when they occurred. Our analyses demonstrate the effects of past spatiotemporal forces on the species' likely paleodistributions, which give us a better understanding of the current fragmented population structure of this endangered plant. Under this framework, we found strong evidence for patterns of range shift and expansion, and population divergence events post‐LGM, resulting in the formation of its five distinct genetic units.

In agreement with the results of Lee et al. (2022), we found a similar number of optimum genetic subdivisions (i.e., *K* = 2 to *K* = 5) but here, we present novel findings by more accurately elucidating the (phylo)genetic relationships of these population groups and lineages. Using multiple independent SNPs, our SNAPP and DIYABC analyses corroborated our STRUCTURE (*K* = 5) results and further characterized the distinctiveness of these population subdivisions in the context of phylogeography (Avise, 2000). We believe that these findings were made possible by the use of multiple independent genealogical samples in the form of SNPs, which phylogeographic studies have taken advantage of due to their accuracy in elucidating phylogenetic relationships and population parameters (Edwards & Beerli, 2000; Felsenstein, 2006; Leaché & Oaks, 2017; Rannala & Yang, 2003; Reitzel et al., 2013).

While the use of molecular data allowed us to identify and refine the (phylo)genetic relationships of infraspecies lineages and populations, the use of ENM helped us discover a possible past LGM refugium and the directions of post‐glacial habitat expansion and reduction events. The time frames of the succeeding Holocene population distribution dynamics were shown to be congruent with at least several climatic and geological events (e.g., oceanic regression‐transgression, monsoonal changes) that affected central KP during the period.

In comparison with other studies that employed the same maximum entropy algorithm on plants with (overlapping) distribution on the KP (Cho et al., 2020; Chung et al., 2018; Jin et al., 2021; Park et al., 2019), the LGM habitat suitability of our focal species appeared to be considerably scaled down to a small area, implying that white forsythia is a less cold‐adapted species relative to other Korean taxa investigated (e.g., Cho et al., 2020; Chung et al., 2018). On the other hand, the bioclimatic predictors with the highest importance and contribution to the niche models represent one of the first environmental data that attempt to explain some of the ecological requirements of this deciduous shrub. In particular, the precipitation amount of the driest month (bio14) may be highly associated with the species' flowering processes after winter, while the maximum temperature of the warmest month (bio5) can be likely related to its fruiting mechanisms in summer. Still, we do not reject the possibility that the present population structure may have been caused by other environmental conditions not covered by ENM (e.g., edaphic properties) and other evolutionary factors not directly tested with the use of molecular data (e.g., genetic drift, natural selection). The inclusion of occurrence reports from North Korea once validated could also help improve future distribution modeling and our knowledge about the species' possibly wider altitudinal range limits.

We also suggest a more in‐depth future analysis of the demographic history of our focal species' five genetic units by sampling more individuals and populations. The challenges to be expected, however, as also for other rare and/or endangered Korean endemics (e.g., Kang et al., 2023; Yun & Oh, 2022), are the little‐known information about the past regional geological and temporal events that may have impacted the present population size and structure of the species (i.e., historical bottlenecks). The effective population sizes of the genetic subdivisions reported here, therefore, should be viewed with caution because possible demographic events that may have occurred after their divergence (e.g., bottleneck and/or expansion) were not taken into account.

We hope that future studies can more closely look into the above‐mentioned recommendations, especially on the different range dynamics among marginal populations. Because of the increasing temperatures that continue to promote the poleward movement of many organisms, the adaptation of species to changing climates is likely to be determined by the response of populations at range margins (Hampe & Petit, 2005). Studies that combine population genomics and ENM can provide new insights into predicting the impacts of climate change on future population dynamics and the creation of climate‐related conservation plans. An interesting subject is to determine whether the northern‐ and easternmost populations are indeed range limits or are still expanding leading‐edge distributions that will continue to migrate.

## AUTHOR CONTRIBUTIONS

**Homervergel G. Ong:** Data curation (equal); formal analysis (lead); investigation (lead); methodology (lead); resources (equal); software (lead); visualization (lead); writing – original draft (lead); writing – review and editing (lead). **Yong‐In Kim:** Investigation (equal); methodology (equal); resources (equal). **Jung‐Hoon Lee:** Data curation (equal); investigation (equal); resources (equal). **Bo‐Yun Kim:** Investigation (supporting); methodology (supporting); resources (equal). **Dae‐Hyun Kang:** Investigation (supporting); resources (supporting). **Eui‐Kwon Jung:** Investigation (supporting); resources (supporting). **Jae‐Seo Shin:** Investigation (supporting); resources (supporting). **Young‐Dong Kim:** Conceptualization (lead); funding acquisition (lead); project administration (lead); supervision (lead); validation (lead).

## CONFLICT OF INTEREST STATEMENT

The authors declare no conflict of interest.

## Supporting information


Appendix S1.

## Data Availability

The data that support the findings of this study are available in the Supporting Information of this article.

## APPENDIX 1

### 1.1

(a) BIC versus the number of clusters, with the lowest inflection point (“elbow”) indicating the best a priori *K* of 9. (b) a‐score optimization suggesting an optimal number of 11 PCs (red dot). Both suggested parameter settings were used for DAPC.

## APPENDIX 2

### 2.1

Δ*K* estimate showing the optimal number of genetic groups at *K* = 2, *K* = 4, and *K* = 5.

## APPENDIX 3

### 3.1

Comparison of eight demographic history models based on posterior probabilities (*y* axis). The direct approach (a) shows Scenario 2 as the top scenario candidate, while the logistic regression (b) suggests Scenario 1 as the top scenario of choice.

## APPENDIX 4

### 4.1

Second PCA plot (see Figure 5 for first) showing a less biased goodness‐of‐fit of Scenario 1 parameters. The observed dataset (yellow circle) is shown at the border of (but not outside) the posterior distribution (black‐outlined green circles) as explained by PC1 (41.7%) and PC2 (24.1%). This second model checking test was run by selecting previously unused combinations of summary statistics, as follow: mean of non‐zero values for single sample statistics (i.e., mean gene diversity (Nei, 1987) across loci with non‐zero values), mean of complete distribution for two sample statistics (i.e., mean *F*_ST_ distances (Weir & Cockerham, 1984), and mean of Nei's distances (Nei, 1972) across all loci between two samples).

## APPENDIX 5

### 5.1

Parameter estimation graphs showing the prior (red curve) and posterior (green curve) distribution of effective population sizes (NA, *N*1, *N*2, *N*3, *N*4, *N*5) and divergence times (*t*4, *t*3, *t*2, *t*1) for the scenario of choice (Scenario 1). Numbers in brackets above each graph represent the median posterior distribution (but see Table S4 for all parameter estimation statistics for the posterior distribution).

## APPENDIX 6

### 6.1

ROC curve showing a high discrimination performance for the Present (1970–2000) model, as indicated by the AUC (0.878) that is higher than the diagonal line of no discrimination (0.5).

## APPENDIX 7

### 7.1

Response curves of white forsythia probability of habitat suitability or occurrence (*y* axis) to (a) bio14—precipitation amount of the driest month (in mm), (b) bio5—maximum temperature of the warmest month (in °C), (c) bio7—annual range of air temperature (in °C), (d) bio13—precipitation amount of the wettest month (in mm), and (e) bio12—annual precipitation amount (in mm).

## APPENDIX 8

### 8.1

Binary map projections of white forsythia suitable (red) and non‐suitable (non‐red) habitats from the LGM (21 kya) to the Present (1970–2000) as computed using five environmental predictors and adjusted using the 10% training presence threshold value (inset).
