# Correction to the article “Approximate Bayesian computation and ecological niche models elucidate the demographic history and current fragmented population distribution of a Korean endemic shrub”

**DOI:** 10.1002/ece3.10830

**Published:** 2023-12-21

Ong, H. G., Kim, Y.‐I., Lee, J.‐H., Kim, B.‐Y., Kang, D.‐H., Jung, E.‐K., Shin, J.‐S., & Kim, Y.‐D. (2023). Approximate Bayesian computation and ecological niche models elucidate the demographic history and current fragmented population distribution of a Korean endemic shrub. Ecology and Evolution, 13, e10792. https://doi.org/10.1002/ece3.10792

Correction to “The Hallym University, Grant/Award Number: H202206730001.” This should have been “The Hallym University, Grant/Award Number: HRF‐202210‐005.”

We apologize for this error.

