# Supplementary material for: Approximate Bayesian computation and ecological niche models elucidate the demographic history and current fragmented population distribution of a Korean endemic shrub
Source: Ecol Evol. 2023 Dec 6;13(12):e10792. doi: 10.1002/ece3.10792 (PMC10700048; doi:10.1002/ece3.10792)

**Supporting Information for:**

**Approximate Bayesian computation and ecological niche models elucidate the demographic history and current fragmented population distribution of a Korean endemic shrub**

Homervergel G. Ong^1^, Yong‑In Kim^2^, Jung‑Hoon Lee^2^, Bo‑Yun Kim^3^, Dae-Hyun Kang^4^, Eui‑Kwon Jung^5^, Jae-Seo Shin^5^, Young‑Dong Kim^1,5*^

**SI Table 1.** Population and geographic information of white forsythia sampled sites and occurrences in South Korea.

| **Occurrence** | **N** | **Population** | **Genetic group** | **Province** | **Town/City, Village** | **Collection/Voucher number** | **Latitude** | **Longitude** | **Elev** |
| --- | --- | --- | --- | --- | --- | --- | --- | --- | --- |
| 1 | 14 | BA-NM370a | SOUTHERN | Jeollabukdo | Buan, Junggye-ri | KBY2019001 | 35.674667 | 126.564667 | 22 |
| 2 | 12 | BA-NM370b | SOUTHERN | Jeollabukdo | Buan, Cheongrim-ri | KBY2019398 | 35.655833 | 126.612833 | 55 |
| 3 | 14 | AD | EASTERN | Gyeongsangbukdo | Andong, Doro-ri | KBY2019051 | 36.511833 | 128.76625 | 98 |
| 4 | 13 | YJ | NORTHERN | Gyeonggido | Yeoju, Jungam-ri | KBY2019026 | 37.35315 | 127.711233 | 110 |
| 5 | 14 | YD | LOWER-CENTRAL | Chungcheongbukdo | Yeongdong, Seolgye-ri | KBY2019209 | 36.1875 | 127.78215 | 106 |
| 6 | 15 | YD-NM364 | LOWER-CENTRAL | Chungcheongbukdo | Yeongdong, Maecheon-ri | KBY2019235 | 36.170333 | 127.784317 | 126 |
| 7 | 15 | OC | LOWER-CENTRAL | Chungcheongbukdo | Okcheon, Woljeon-ri | KBY2019259 | 36.306333 | 127.55395 | 110 |
| 8 | 14 | GS-NM147 | UPPER-CENTRAL | Chungcheongbukdo | Goesan, Songdeok-ri | KBY2019158 | 36.795617 | 127.93795 | 185 |
| 9 | 13 | GS-NM221 | UPPER-CENTRAL | Chungcheongbukdo | Goesan, Yulji-ri | KBY2019184 | 36.771733 | 127.867833 | 154 |
| 10 |  |  |  | Chungcheongbukdo | Goesan, Chujeom-ri | HGO2019-Ad-10 | 36.862889 | 127.947333 | 132 |
| 11 |  |  |  | Chungcheongbukdo | Goesan, Samsong-ri | ESJeon52427 | 36.6225 | 127.923306 | 333 |
| 12 |  |  |  | Chungcheongbukdo | Jincheon, Geumgok-ri | KBY2019106 | 36.8533 | 127.530583 | 67 |
| 13 |  |  |  | Chungcheongbukdo | Jincheon, Yongjeong-ri | HGO2022-Ad-13 | 36.843444 | 127.526333 | 76 |
| 14 |  |  |  | Chungcheongbukdo | Jincheon, Yongjeong-ri | HGO2022-Ad-14 | 36.834944 | 127.534528 | 107 |
| 15 |  |  |  | Chungcheongbukdo | Jincheon, Yongjeong-ri | HGO2022-Ad-15 | 36.8305 | 127.520667 | 80 |
| 16 |  |  |  | Chungcheongbukdo | Jincheon, Yeondam-ri | HGO2022-Ad-16 | 36.790694 | 127.483028 | 91 |
| 17 |  |  |  | Chungcheongbukdo | Jincheon, Gugok-ri | 희귀특산2013-044/KHB1434468 | 36.837056 | 127.487972 | 150 |
| 18 |  |  |  | Chungcheongbukdo | Jincheon, Ogap-ri | 충북-10/KHB359663 | 36.840194 | 127.501817 | 74 |
| 19 |  |  |  | Chungcheongbukdo | Chungju, Bonghwang-ri | NGH60583 | 37.055783 | 127.830967 | 96 |
| 20 |  |  |  | Jeollabukdo | Buan, Junggye-ri | 040519-230 | 35.633 | 126.579111 | 68 |
| 21 |  |  |  | Gyeongsangbukdo | Uiseong, Uiseong-eup | KBY2019077 | 36.341111 | 128.695678 | 178 |
| 22 |  |  |  | Gangwondo | Wonju, Donghwa-ri | KYI2021-Ad-22 | 37.33595 | 127.84795 | 97 |

**SI Note 1.** Eight white forsythia demographic history scenarios, model assumptions, and hypothetical basis.

**Model Assumptions**

Eight alternative scenarios (categorized in two main groupings) explaining possible white forsythia demographic history events are here described. All models were constructed based on the patterns of the central-marginal hypothesis, as observed in our earlier study (Lee et al., 2022), wherein genetic diversity among populations declined from a central region toward the periphery. Each of these models are made up of five genetic groups identified and supported by the results of our (phylo)genetic clustering analyses in DAPC, STRUCTURE (K = 5), and SNAPP. The tree topology followed those of the results in SNAPP. The five genetic groups comprise the following: SOUTHERN (BA-NM370a and BA-NM370b), NORTHERN (YJ), EASTERN (AD), UPPER-CENTRAL (GS-NM147 and GS-NM221), and LOWER-CENTRAL (OC, YD and YD-NM364). In this study, we hypothesize that the most probable demographic history, which eventually formed the five genetic subdivisions, is via a series of divergence events from one of the two subcentral groups, hence the two categories of models (see the two categories below).

In all eight models, it is assumed that during the earliest divergence time (t4), an ANCESTRAL POPULATION (NA) was split into two major lineages: a SOUTHERN (N1/Pop 1) lineage, which remained isolated since split, and a large central metapopulation lineage, which eventually gave rise to other remaining genetic groups. Two putative central metapopulation lineages are here proposed and tested: those of the UPPER-CENTRAL (N3/Pop 3) and the LOWER-CENTRAL (N2/Pop 2) groups. It is assumed that most, if not all population expansion and divergence (or admixture) events that came after t4, occurred from either of the two central groups. However, it is clarified that unlike the UPPER-CENTRAL lineage (N3/Pop 3), the LOWER-CENTRAL lineage (N2/Pop 2) may not have given rise to the NORTHERN group (N5/Pop 5) due to geographical limitations (see Figure 1). Also, in other scenarios, it is proposed that the NORTHERN group (N5/Pop 5) may have branched out from the expansion or split of the EASTERN group (N4/Pop 4), but not vice versa.

**A. UPPER-CENTRAL Divergence Models (Scenarios 1–4)**

**Scenario 1** (Sequential UPPER-CENTRAL to EASTERN and NORTHERN expansion or split, late LOWER-CENTRAL divergence)

At t4, after the split of the SOUTHERN group (N1/Pop 1) and the UPPER-CENTRAL lineage from the ANCESTRAL POPULATION (NA), the UPPER-CENTRAL lineage sequentially gave rise to the EASTERN group (N4/Pop 4) at t3, and then to the NORTHERN group (N5/Pop 5) at t2. Finally, the LOWER-CENTRAL group (N2/Pop 2) was characterized from the UPPER-CENTRAL lineage (N3/Pop 3) after its divergence from the latter at t1.

**Scenario 2** (Sequential UPPER-CENTRAL to NORTHERN and EASTERN expansion or split, late LOWER-CENTRAL divergence)

At t4, after the split of the SOUTHERN group (N1/Pop 1) and the UPPER-CENTRAL lineage from the ANCESTRAL POPULATION (NA), the UPPER-CENTRAL lineage sequentially gave rise to the NORTHERN group (N5/Pop 5) at t3, and then to the EASTERN group (N4/Pop 4) at t2. Finally, the LOWER-CENTRAL group (N2/Pop 2) was characterized from the UPPER-CENTRAL lineage (N3/Pop 3) after its divergence from the latter at t1.

**Scenario 3** (Early UPPER-CENTRAL and EASTERN split, EASTERN to NORTHERN expansion or split, late LOWER-CENTRAL and UPPER-CENTRAL divergence)

At t4, after the split of the SOUTHERN group (N1/Pop 1) and the UPPER-CENTRAL lineage from the ANCESTRAL POPULATION (NA), the UPPER-CENTRAL lineage at t3, gave rise to the EASTERN group (N4/Pop 4), from which the NORTHERN group (N5/Pop 5) branched out at t2. Finally, the LOWER-CENTRAL group (N2/Pop 2) was characterized from the UPPER-CENTRAL lineage (N3/Pop 3) after its divergence from the latter at t1.

**Scenario 4** (Early UPPER-CENTRAL and LOWER-CENTRAL split, UPPER-CENTRAL to EASTERN expansion or split, late EASTERN and NORTHERN divergence)

At t4, after the split of the SOUTHERN group (N1/Pop 1) and the UPPER-CENTRAL lineage from the ANCESTRAL POPULATION (NA), the UPPER-CENTRAL lineage gave rise to the LOWER-CENTRAL group (N2/Pop 2) at t3. The UPPER-CENTRAL lineage (N3/Pop 3) at t2, then gave rise to the EASTERN group (N4/Pop 4), from which the NORTHERN group (N5/Pop 5) branched out at t1.

**B. LOWER-CENTRAL Divergence Models (Scenarios 5–8)**

**Scenario 5** (Early LOWER-CENTRAL and UPPER-CENTRAL split, UPPER-CENTRAL to EASTERN expansion or split, late EASTERN and NORTHERN divergence)

At t4, after the split of the SOUTHERN group (N1/Pop 1) and the LOWER-CENTRAL lineage from the ANCESTRAL POPULATION (NA), the LOWER-CENTRAL lineage (N2/Pop 2) gave rise to the UPPER-CENTRAL group (N3/Pop 3) at t3. The UPPER-CENTRAL group (N3/Pop 3) at t2, then gave rise to the EASTERN group (N4/Pop 4), from which the NORTHERN group (N5/Pop 5) branched out at t1.

**Scenario 6** (Early LOWER-CENTRAL and EASTERN split, LOWER-CENTRAL to UPPER-CENTRAL expansion or split, late EASTERN and NORTHERN divergence)

At t4, after the split of the SOUTHERN group (N1/Pop 1) and the LOWER-CENTRAL lineage from the ANCESTRAL POPULATION (NA), the LOWER-CENTRAL lineage gave rise to the EASTERN group (N4/Pop 4) at t3. The UPPER-CENTRAL group (N3/Pop 3) was then characterized from the LOWER-CENTRAL lineage (N2/Pop 2) after its divergence from the latter at t2. Finally, the NORTHERN group (N5/Pop 5) branched out from the EASTERN group (N4/Pop 4) at t1.

**Scenario 7** (Early LOWER-CENTRAL and EASTERN split, EASTERN to NORTHERN expansion or split, late LOWER-CENTRAL and UPPER-CENTRAL divergence)

At t4, after the split of the SOUTHERN group (N1/Pop 1) and the LOWER-CENTRAL lineage from the ANCESTRAL POPULATION (NA), the LOWER-CENTRAL lineage at t3, gave rise to the EASTERN group (N4/Pop 4), from which the NORTHERN group (N5/Pop 5) branched out at t2. Finally, the UPPER-CENTRAL group (N3/Pop 3) was characterized from the LOWER-CENTRAL group (N2/Pop 2) after its divergence from the latter at t1.

**Scenario 8** (Early LOWER-CENTRAL and EASTERN split, UPPER-CENTRAL admixture, late EASTERN and NORTHERN divergence)

At t4, after the split of the SOUTHERN group (N1/Pop 1) and the LOWER-CENTRAL lineage from the ANCESTRAL POPULATION (NA), the LOWER-CENTRAL lineage gave rise to the EASTERN group (N4/Pop 4) at t3. At t2, the UPPER-CENTRAL group (N3/Pop 3) admixture was a result of a secondary contact between the LOWER-CENTRAL lineage (N2/Pop 2) and the EASTERN group (N4/Pop 4), with admixture rate relative to the latter. Finally, the NORTHERN group (N5/Pop 5) branched out from the EASTERN group (N4/Pop 4) at t1.

**SI Table 2.** Prior parameter settings used in DIYABC

| **Parameter** | **Type** | **Prior** |
| --- | --- | --- |
| N1 | N | Uniform (10–8000) |
| N2 | N | Uniform (10–10000) |
| N3 | N | Uniform (10–14000) |
| N4 | N | Uniform (10–3000) |
| N5 | N | Uniform (10–5000) |
| NA | N | Uniform (400000–600000) |
| t1 | t | Uniform (10–2000) |
| t2 | t | Uniform (10–3000) |
| t3 | t | Uniform (10–5000) |
| t4 | t | Uniform (10–10000) |
| ra | r | Uniform (0.001–0.999) |

N1, N2, N3, N4 and N5 represent the effective population sizes of the five genetic groups; NA represents the ancestral effective population size; t1, t2, t3 and t4 represent the time of demographic events converted into number of generations.

**SI Table3.** Type I error showing the rejection of Scenario 1 about 90% of the time in both the direct (87.5%) and logistic regression (92.3%) approach despite being the true scenario. Hence, Scenario 1 shows a higher confidence as the scenario of choice than Scenario 2. Only the first and last 10 pseudo-observed datasets are displayed.


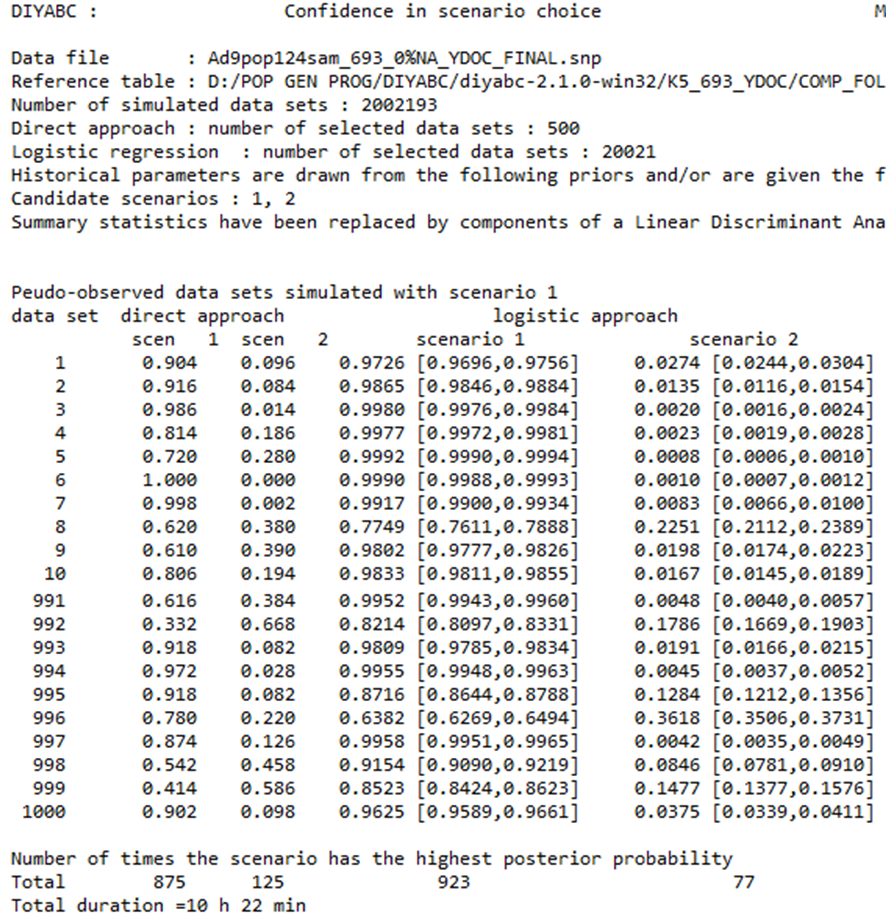


**SI Table4.** Parameter estimation statistics (mean, median, mode, quantiles) of the posterior distribution for Scenario 1. Reported and recalibrated values in the manuscript are those of the mean.


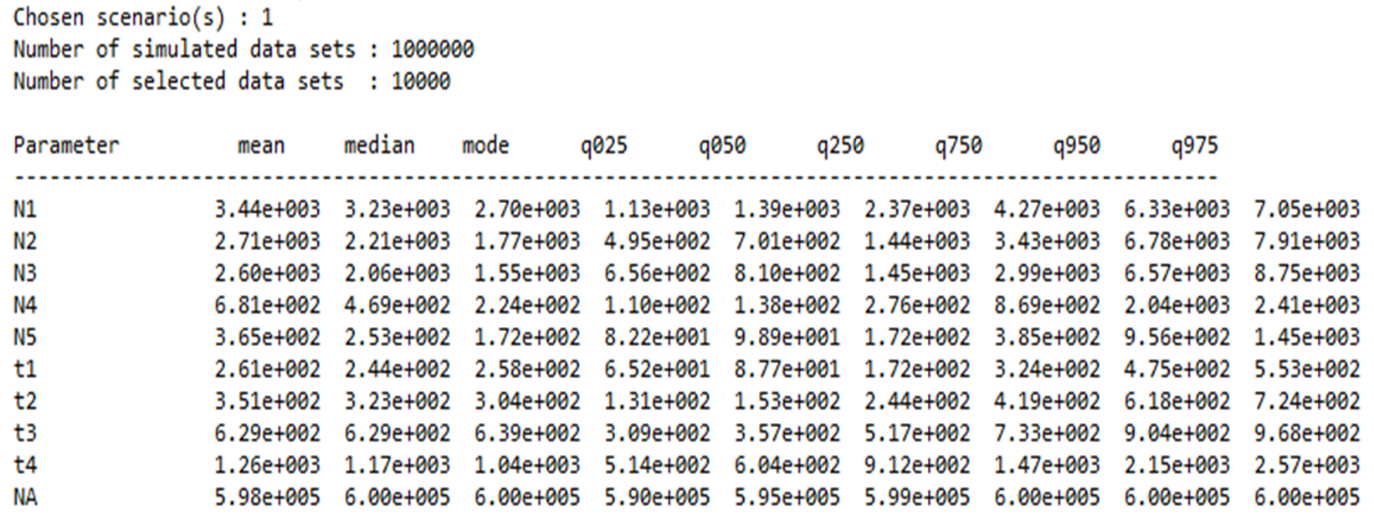

Supplement: Supplementary file 1 — Appendix S1. [file ECE3-13-e10792-s001.docx]
